# Usefulness of low dose chest CT for initial evaluation of blunt chest trauma: Erratum

**DOI:** 10.1097/MD.0000000000007234

**Published:** 2017-06-08

In the article “Usefulness of low dose chest CT for initial evaluation of blunt chest trauma”,^[1]^ which appeared in Volume 96, Issue 2 of *Medicine*, “This work was supported by (2012) grant from Department of Medical Sciences, The Graduate School, Ajou University” should have appeared in the article.
